# Optic Neuritis in Resolving Phase of COVID-19 Infection and Its Management: A Case Report

**DOI:** 10.7759/cureus.58257

**Published:** 2024-04-14

**Authors:** Aanchal Singh, Swapneel Mathurkar

**Affiliations:** 1 Ophthalmology, Datta Meghe Institute of Higher Education and Research, Wardha, IND

**Keywords:** diminution of vision, optic neuritis, retinopathy, visual acuity, refractive error, neuropathy

## Abstract

Optic neuritis is assumed to be immune-mediated, although the specific antigens that cause demyelination are uncertain. Systemic T-cell activation is detected at the onset of symptoms, which occurs before alterations in cerebrospinal fluid (CSF). The optic nerve disease is a rare disease and can occur in one or both eyes, especially in those with no established inflammatory or autoimmune illnesses. Adult ophthalmic neuritis is usually unilateral and is frequently associated with multiple sclerosis (MS). Generally, it starts as a rapid loss of vision and pain in eye movement. It progresses and achieves the maximal deficiency over a week. The objectives of this paper were to determine the association between coronavirus disease 2019 (COVID-19) and optic neuritis and to study the management of optic neuritis in the resolving phase of COVID-19. A case study was done on a 38-year-old female complaining of sudden diminution of vision in her right eye for one week. She tested positive on the reverse transcriptase-polymerase chain reaction (RT-PCR) test for COVID-19 for which she was managed symptomatically and was started on antiretrovirals. This case report is based on an infrequent COVID-19 complication. It has been proposed that this virus has the probability of manifesting various neurological complications. In our case, optic neuritis occurs mainly three weeks after COVID-19 infection. Our patient was managed by intravenous methylprednisolone injection followed by oral prednisone for 14 days. So, further case studies will be required to support the above treatment plan for optic neuritis caused by the severe acute respiratory syndrome coronavirus 2 (SARS-CoV-2). Unilateral or bilateral optic neuritis can occur as a neurological complication in the resolving stage of COVID-19 infection. Early detection and treatment with steroids can result in the best visual outcome.

## Introduction

The new severe acute respiratory syndrome coronavirus 2 (SARS-CoV-2) causing coronavirus disease 2019 (COVID-19) has broadly similar disease presentation to respiratory illness. Moreover, there are recognized cases of neuro-ophthalmic disease along with the setting of SARS-CoV-2 infection, including a range of disorders like optic neuritis, Miller Fisher syndrome, cranial neuropathy with vision loss, and neuromyelitis optic. Optic neuritis is inflammation of the optic nerve, which has numerous origins [1]. The virus could overstate the nervous system through a variety of mechanisms: one is directly through neurotoxicity; the other is that the virus, after reaching the nervous system over different routes, binds to receptors of angiotensin-converting enzyme 2 (ACE2), the blood-brain barrier, and by increasing blood clotting along with impairing blood clot formation [2]. Optic neuritis is usually unilateral and can go along with acute and severe eye pain or pain in the periorbital region, reaching its extreme manifestations in seven days [3]. Optic neuritis is assumed to be immune-mediated, although the specific antigens that cause demyelination are uncertain. Systemic T-cell activation is detected at the onset of symptoms, which occurs before alterations in cerebrospinal fluid (CSF). Activation of T-cells causes cytokines and other inflammatory chemicals to be released [4]. Although activation of B-cells contrary to myelin essential protein is not visible in the peripheral blood, it can be detected in the CSF of patients with optic neuritis [5]. There is a rare occurrence of optic nerve disease, which can occur in one or both eyes, especially in those with no established inflammatory or autoimmune illnesses. Adult ophthalmic neuritis is usually unilateral and is frequently associated with multiple sclerosis (MS). Generally, it starts as a rapid loss of vision and pain in the eye movement. It is progressive and achieves maximal deficiency over a week. The diagnosis is usually made clinically based on the patient's medical history and clinical findings. In most situations, images of the brain and the orbit of the eye taken by magnetic resonance imaging (MRI) aid in the diagnosis. Planning the line of treatment is usually uncomplicated after confirmation.

Coronavirus is a ribonucleic acid (RNA) virus affecting humans and birds [6]. Acute respiratory tract infections in farmed chickens were primarily observed in the 1930s, leading to the findings associated with coronavirus [7]. In 1960, a youngster infected with the human coronavirus in the United Kingdom developed common cold-like symptoms [8]. It has caused mild to severe illnesses since its discovery. COVID-19 has resulted in high mortality and morbidity, as well as affecting a variety of organs. Ocular symptoms, on the other hand, have been documented rarely. Acute optic neuritis commencing bilaterally is a rare manifestation, predominantly in those with no established autoimmune or inflammatory conditions. Adult optic neuritis is typically unilateral and frequently associated with MS. It usually starts with a sudden onset of loss of vision with pain in the eye movement and advances over the passage of a week to achieve its extreme medical appearance [9].

## Case presentation

**Figure 1:**
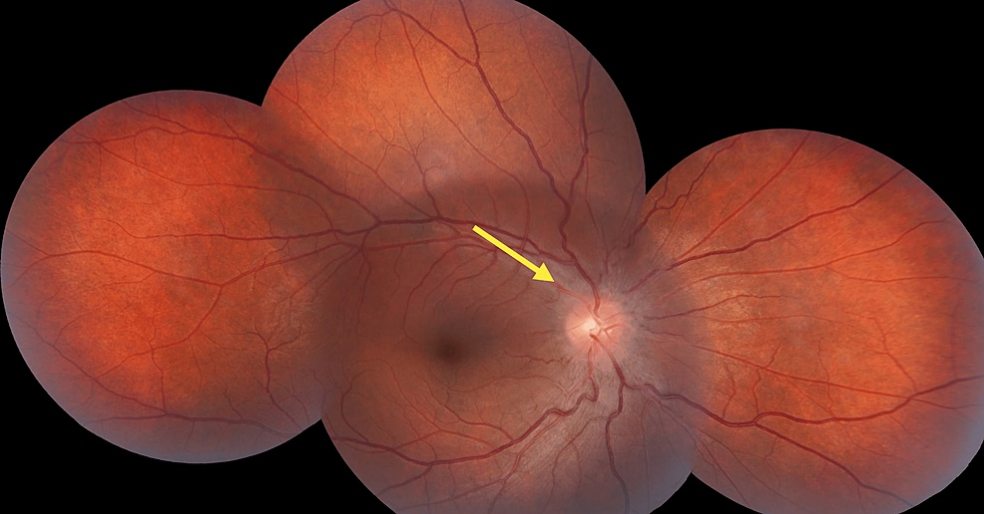
Fundus photography during treatment (right eye) The right eye showed an expansion of the blind spot.

**Figure 2:**
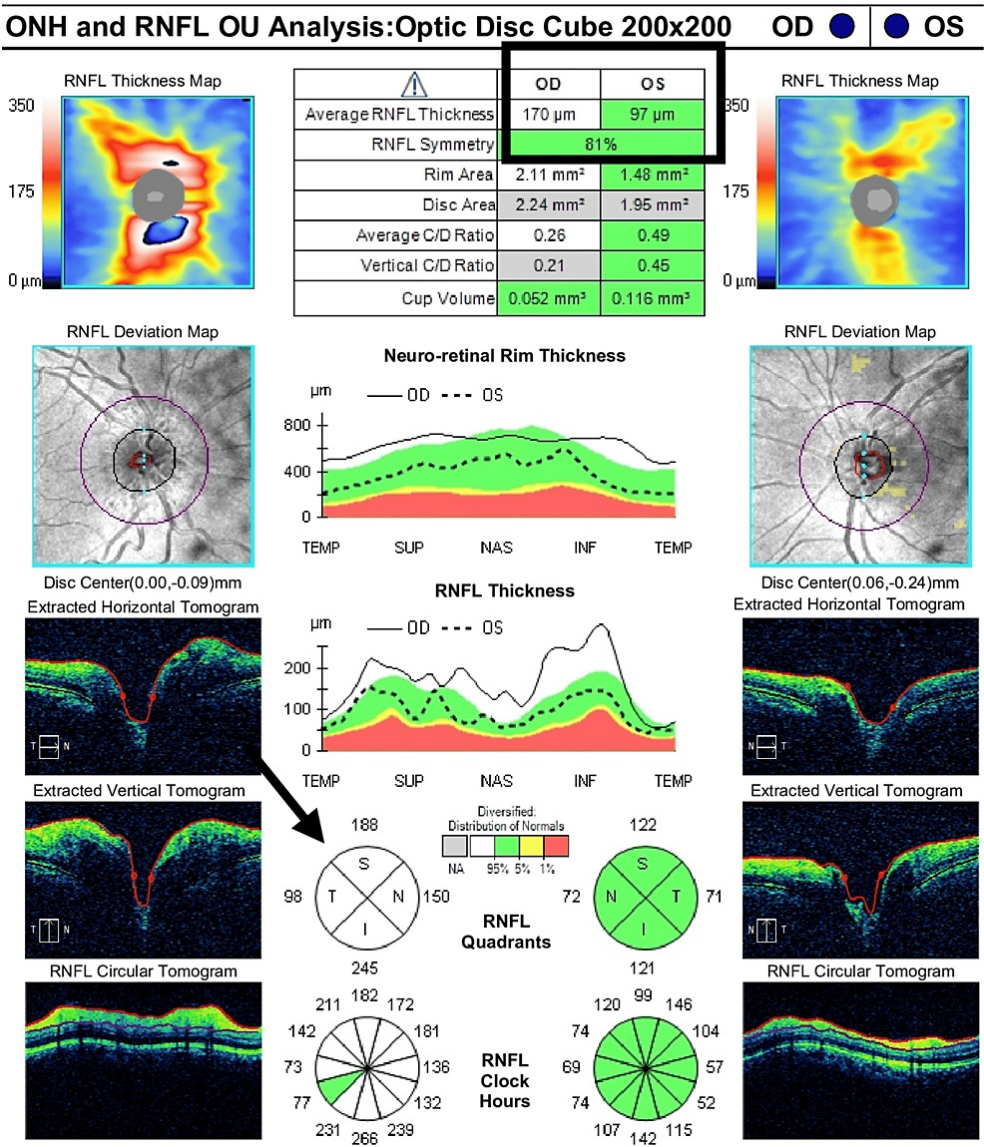
Optic disc edema identified on OCT before treatment (right eye) The brain and orbit scan with contrast showed a slightly enhanced and thickened intraorbital part of the right optic nerve, suggestive of optic neuritis. OCT showed optical disc edema with an average RNFL thickness of 170 micrometers. OCT: Optical coherence tomography; RNFL: Retinal nerve fiber layer; ONH: Optic nerve hypoplasia; OU: Oculus uterque

A 38-year-old woman presented to the Ophthalmology Outpatient Section with a primary complaint of sudden diminishment of vision in her right eye for the past week. The patient had a four-day history of pain on upward and downward movements in the right eye and a history of fever and generalized weakness one month ago (COVID-19 RT-PCR positive one month ago). Her vitals were stable in the general examination within normal limits. On ocular examination, the right eye's visual acuity came from counting fingers at one meter, which didn't improve with pinhole, which gave evidence of relative afferent pupillary defect (RAPD). The anterior segment appeared to be within normal limits. On examination of the fundus, the right eye's retina showed optic disc edema having a usual thickness of 233 micrometers with congestion along with alteration of the optical disc cup (0.21-0.25). In the right eye, color visualization and contrast sensitivity were also affected. In the left eye examination, visual acuity was 6/6. The anterior and posterior segments were within normal limits. In the left eye, color vision and contrast sensitivity were expected. On perimetry, the right eye showed an expansion of the blind spot and the left eye showed a standard blind spot (Figure 1). On blood investigation, random blood sugar was 118 mg/dl, and kidney function tests and liver function tests were within normal limits. On MRI, the brain and orbit scan with contrast showed a slightly enhanced and thickened intraorbital part of the right optic nerve, suggestive of optic neuritis. Optical coherence tomography (OCT) showed optical disc edema with an average retinal nerve fiber layer (RNFL) thickness of 170 micrometers (Figure 2).

**Figure 3:**
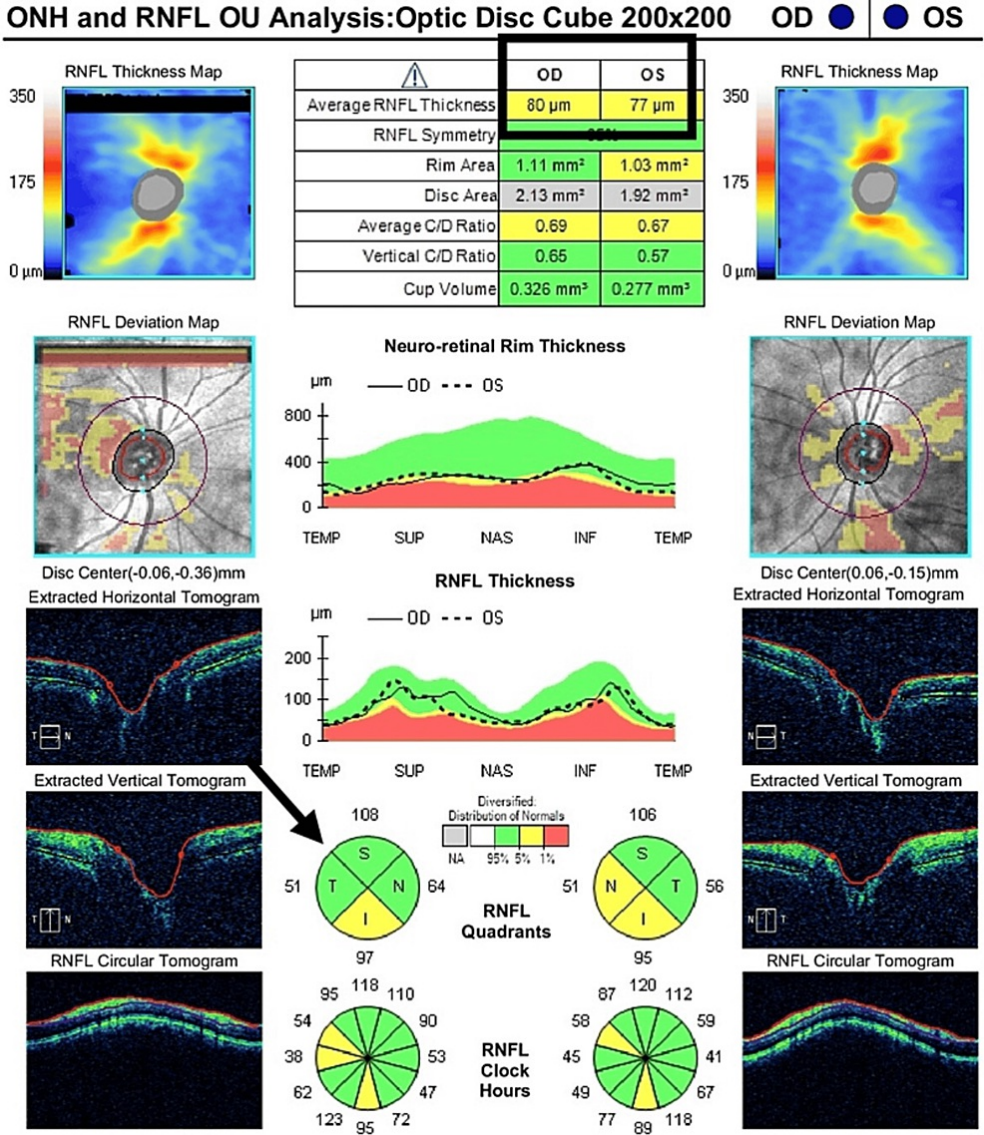
Optic disc edema identified on OCT after treatment (right eye) Right optic disc edema was in the revolving stage (average RNFL thickness of 80 micrometers). OCT: Optical coherence tomography; RNFL: Retinal nerve fiber layer; ONH: Optic nerve hypoplasia; OU: Oculus uterque

**Figure 4:**
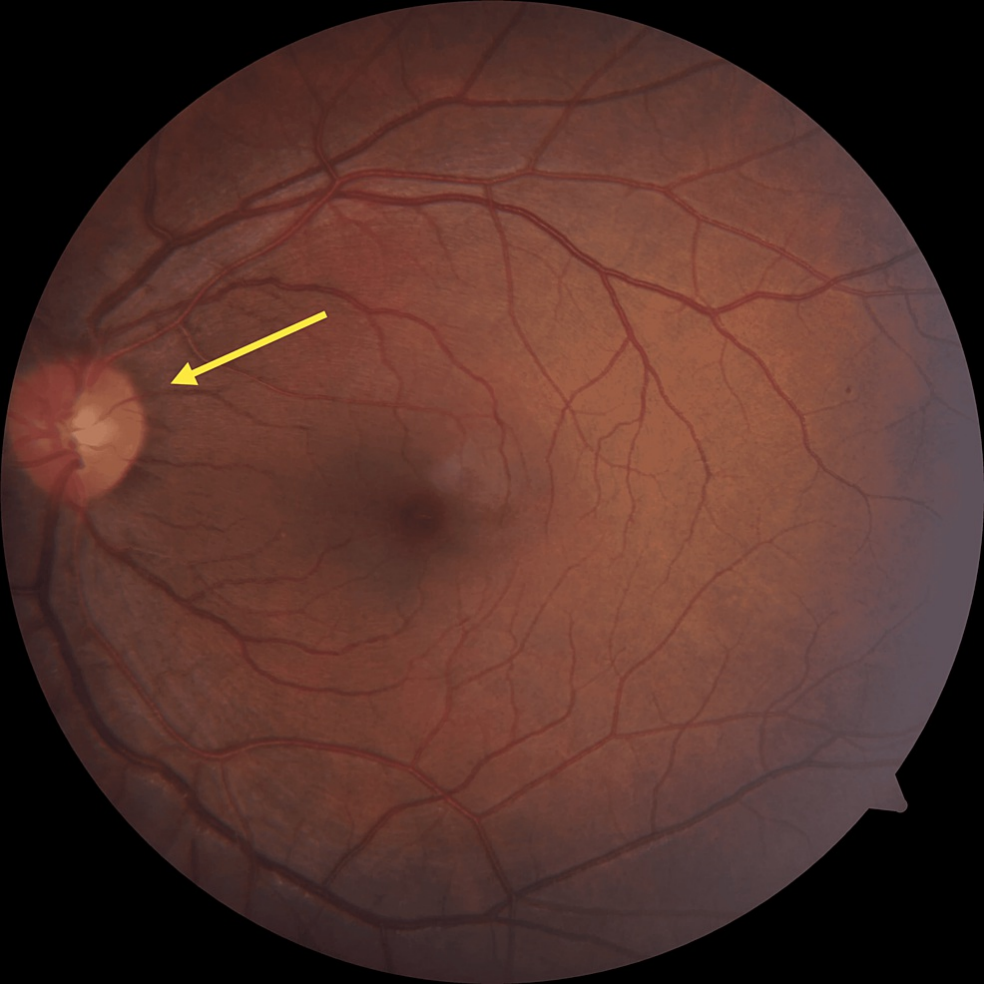
Fundus photography after treatment (right eye) On day fifteen, the right optic disc was found to have standard margins, size, shape, and mild pallor with still affected color vision and contrast sensitivity with visual acuity improved to 6/9.

Treatment started with injections of methylprednisolone (1 g) intravenous for three days, followed by tablets of prednisolone on a tapering dose for 11 days. On day four, right optic disc edema was in the revolving stage (average RNFL thickness of 80 micrometers) (Figure 3). Color vision and contrast sensitivity were still affected. Visual acuity enhanced from finger counting to 6/24. On day fifteen, the right optic disc was found to have standard margins, size, shape, and mild pallor with still affected color vision and contrast sensitivity with visual acuity improved to 6/9 (Figure 4). 

## Discussion

Optic neuritis is usually unilateral, accompanied by acute eye or periorbital pain, and accomplishes its highest manifestation within a week. Optic neuritis is immune-mediated, though the specific antigens responsible for causing demyelination are uncertain. At the onset of symptoms, systemic T-cell activation occurs before alterations in the CSF. T-cell activation causes cytokines and other inflammatory chemicals. The analysis relies on the patient's medical history, ocular examination, OCT, and MRI [10]. The treatment should be started as early as possible. Intravenous methylprednisolone and oral prednisolone altered according to the patient's age, medical history, and interaction with different drugs are the typical treatment plans for optic neuritis [11]. The infection condition of the eye's nerves, demyelinating illness, results in an abrupt end of visual effect in one eye. It is strongly linked to MS and is present in 16-19% of patients, with 51% experiencing it at some point during the infection stay. Optic neuritis is immune-mediated but the antigens that cause demyelination are unknown. Systemic T-cell activation occurs at the outset of symptoms before alterations in the CSF. The excretion of cytokines and other infectious chemicals occurs when T-cells are activated. B-cell initiation against myelin's essential protein is visible in the CSF of patients with nerve damage to the eyes. Initially, antibodies to the myelin oligodendrocyte glycoprotein played a role in MS. Some research revealed it as a separate infection known as myelin oligodendrocyte glycoprotein antibody-associated disease (MOGAD), manifesting as nerve defects in the eyes, acute disseminated encephalomyelitis, and transverse myelitis. Neuromyelitis optica antibodies (aquaporin-4 autoantibodies) are detected and pathologically characterized as astrocytopathies. In MOGAD disease, the optic nerve is more typically damaged and is more likely to be bilaterally involved. The patient had an acute infection of the nerves of the eyes caused by SARS-CoV-19, by exclusion. In the mentioned case, there is the presentation of comparatively rare COVID-19 complications. It has been proposed that this virus has the probability of manifesting various neurological complications. In our case, optic neuritis occurs mainly after three weeks post COVID-19 infection, and the case was properly managed by intravenous methylprednisolone injection followed by oral prednisone for fourteen days. So, a few further case studies will be required to support the above treatment plan for optic neuritis caused by the SARS-CoV-2 virus.

## Conclusions

Unilateral or bilateral optic neuritis can occur in the resolving stage of COVID-19 infection as a neurological complication. Early detection and treatment with steroids can result in the best visual outcome. On presentation, the patient showed the same effects that are characteristic of acute infection of the nerves of the eyes. MRI scans of the brain and orbits and lumbar puncture to rule out MS or another genetic disease were not positive, as were other clinical tests. COVID-19 viral infection has a global impact as a pandemic, and its scope as a clinical condition is still little understood and described.
